# A Review on Thermal Properties of Hydrogels for Electronic Devices Applications

**DOI:** 10.3390/gels9010007

**Published:** 2022-12-23

**Authors:** Fei Xin, Qiang Lyu

**Affiliations:** 1 Key Laboratory of Ministry of Education for Electronic Equipment Structure Design, Xidian University, Xi’an 710071, China; 2 State Key Laboratory of Multiphase Flow in Power Engineering, Xi’an Jiaotong University, Xi’an 710049, China

**Keywords:** hydrogels, electronic devices, thermal stability, thermoresponsiveness, thermal conductivity

## Abstract

Hydrogels, as a series of three-dimensional, crosslinked, hydrophilic network polymers, exhibit extraordinary properties in softness, mechanical robustness and biocompatibility, which have been extensively utilized in various fields, especially for electronic devices. However, since hydrogels contain plenty of water, the mechanical and electrochemical properties are susceptible to temperature. The thermal characteristics of hydrogels can significantly affect the performance of flexible electronic devices. In this review, recent research on the thermal characteristics of hydrogels and their applications in electronic devices is summarized. The focus of future work is also proposed. The thermal stability, thermoresponsiveness and thermal conductivity of hydrogels are discussed in detail. Anti-freezing and anti-drying properties are the critical points for the thermal stability of hydrogels. Methods such as introducing soluble ions and organic solvents into hydrogels, forming ionogels, modifying polymer chains and incorporating nanomaterials can improve the thermal stability of hydrogels under extreme environments. In addition, the critical solution temperature is crucial for thermoresponsive hydrogels. The thermoresponsive capacity of hydrogels is usually affected by the composition, concentration, crosslinking degree and hydrophilic/hydrophobic characteristics of copolymers. In addition, the thermal conductivity of hydrogels plays a vital role in the electronics applications. Adding nanocomposites into hydrogels is an effective way to enhance the thermal conductivity of hydrogels.

## 1. Introduction

Nowadays, malleable and bendable flexible electronic devices with high efficiency and low cost have been extensively used in the areas of information storage, energy conversion, medical devices, national defense construction, etc. [1] The selection of materials is crucial for flexible electronic devices. Hydrogels, as a series of 3D insoluble polymers with crosslinked hydrophilic networks, show outstanding performance in absorbing water [2,3,4]. Many hydrophilic functional groups attach to the polymer backbone of hydrogels, which leads to the excellent swelling ability of hydrogels [5]. Hydrogels are in the intermediate state between solid and liquid, which shows remarkable solid-like plasticity and fluid-like mobility. They possess the merits of low elastic modulus, tunable physical characteristics, high adaptability, fast stimulus responsiveness and excellent biocompatibility, which can be promising materials for the growth of flexible electronics [2,6,7]. Hydrogels can be used as the base materials of flexible sensors, actuators, transistors, capacitors, batteries, ionic skin, touch panels, drug release controllers for the areas of wearable system, energy harvesting and storage systems, medical treatment, etc.

Hydrogels, with their excellent biocompatibility, can not only adhere to the organ surface firmly without falling off from the organ, but also closely imitate the mechanical, chemical and optical characteristics of biological tissues compared with traditional inorganic materials, which is especially appealing for biological applications, such as wearable devices, drug delivery, biomedical implantation, wound dressings, soft robotics, etc. [8]. Flexible wearable sensing devices can closely attach to the skin surface and continuously monitor human movement and physiological information, which is expected to bring more tremendous changes to people’s lives [9].

However, since hydrogels contain plenty of water, the mechanical and electrochemical properties are very sensitive to temperature. Additionally, the implementation of hydrogel functionality usually depends on temperature. Thermal properties such as the thermal stability, thermal responsiveness and thermal conductivity of hydrogels are crucial for the real-world flexible electronic devices. Hydrogels, with a high water content, usually freeze under low temperatures and can easily dry out under high temperatures. Improving the thermal stability of hydrogels under different working temperatures is necessary for hydrogels to adapt to extreme environments. Furthermore, some functional hydrogels are so sensitive to temperature changes that they undergo discontinuous shape, appearance or size change over a small temperature range. These kinds of hydrogels can be utilized to produce multifunctional parts for flexible electronic devices. In addition, polymer materials have been widely used for electronic packaging. Hydrogels, as one type of polymer materials with low cost, easy production, fine insulation and viscosity are attractive. However, the thermal conductivity of hydrogels, referring to the heat transfer rate, is generally low. With the improved requirement for the thermal design of electronic devices with high heat flux, hydrogels with high thermal conductivity are needed. Hence, much attention should be paid to understanding and enhancing the thermal properties of hydrogels.

**Figure 1:**
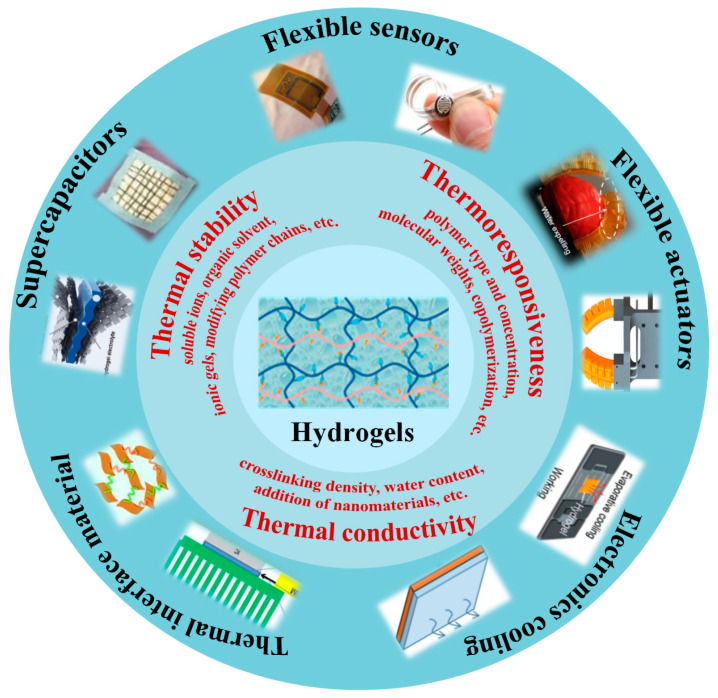
Main thermal characteristics and applications of hydrogels in electronics.

In this review, the thermal properties of hydrogels with the applications in electronic devices are analyzed systematically. The main thermal characteristics with the applications of hydrogels in electronic devices are shown in Figure 1. The research on hydrogel thermal stability, thermoresponsiveness and thermal conductivity from scholars is discussed in detail.

## 2. Thermal Stability of Hydrogels with Applications in Electronic Devices

### 2.1. Thermal Stability of Hydrogels with Enhancement Methods

Due to the containment of a large amount of water, hydrogels are prone to losing mechanical toughness and become hard and brittle, under low temperatures with water freeze and high temperatures with water loss. Meanwhile, due to the freeze or loss of water in hydrogels, some other excellent properties can also be lost, including electrical conductivity [10,11], transparency [12,13] and self-repairability [14,15], under these extreme environments, which severely limits the application and development of hydrogels. Even under normal ambient temperatures, the continuous evaporation of moisture inside hydrogels means they are not usable long-term and eventually lose their original performance. Therefore, it is necessary for hydrogels to adapt to the environment and improve the thermal stability, for the purpose of presenting high temperature-independent mechanical behaviors and excellent anti-freezing and anti-drying properties. To solving such a problem, researchers have conducted lots of work.

#### 2.1.1. Anti-Freezing Properties

Water-based hydrogels can inevitably freeze, become hard and brittle and lose electrical conductivity at sub-zero temperatures. Reducing the solid-liquid transition temperature of hydrogels is urgently required to expand their application range at low temperatures [16]. At present, researchers have proposed various methods to improve the anti-freezing capability of hydrogels, such as introducing soluble ions in solvents [17], introducing organic solvent into hydrogels [18,19], forming ionic gels by replacing aqueous solutions with ionic liquid [20,21] and modifying polymer chains [22,23]. Table 1 presents the general methods proposed by researchers to enhance the anti-freezing properties of hydrogels.

(1) Introducing soluble ions in solvents

As is well known, salts are helpful in melting ice in winter. When soluble ions are added into water, hydration ions are formed by the interaction between water molecules and soluble ions through ionic interaction. This destroys the standard structure of tetrahedral arrangement formed by the hydrogen bonding of water molecules. As a result, the distance between the water molecules increases, leading to smaller intermolecular force, which lowers the freezing temperature for solidification. Hydrogels contain large numbers of water molecules. Because of the ionic hydration and tunable freezing point of salts, dissolving inorganic salts (such as NaCl, CaCl_2_, KCl and LiCl) in hydrogels can lower the freezing point of hydrogels, preventing hydrogels from losing mechanical toughness and other properties due to freezing at sub-zero temperatures [17,18,19,20].

**Figure 2:**
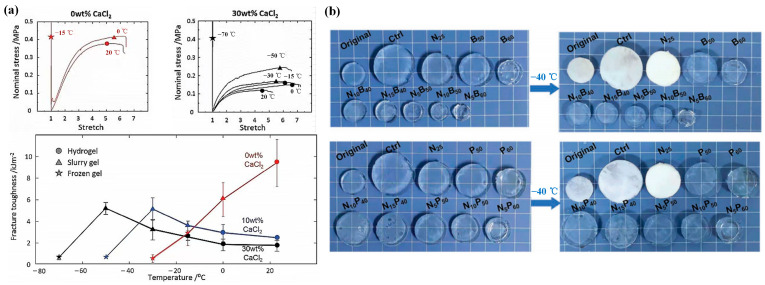
Effects of introducing soluble ions in solvents on the anti-freezing properties of hydrogels: (**a**) Mechanical properties and fracture toughness of hydrogels with changed CaCl_2_ concentration under different temperature; (**b**) Anti-freezing behavior of NH_4_Cl/betaine or NH_4_Cl/proline based hydrogels at −40 °C. Reprinted with permission from [17,26].

Morelle et al. [17] added CaCl_2_ into polyacrylamide-alginate double-network hydrogels. They found that the existence of ionic compounds could effectively decrease the freezing temperature of hydrogels. Ca^2+^ ions in CaCl_2_ could produce strong hydrogen bonding with water molecules, forming the Ca(H_2_O)_n_^2+^ structure. It destroyed the hydrogen bonding of the water molecules, which improved the anti-freezing properties of hydrogels. Figure 2a displayed the variation of stress with stretch for the 0, 10, and 30 wt% CaCl_2_ gels under different working temperature. It could be seen that a higher CaCl_2_ concentration results in better anti-freezing properties of hydrogels. The hydrogels could still maintain high stretchability and fracture toughness under the extremely cold condition of −57 °C. Li et al. [24] developed sustainable and inexpensive electrolyte hydrogel surfaces through introducing the ions of KCl, NaCl or CaCl_2_ into the hydrogel matrix to prevent freezing. They found that the melting point of hydrogels decreased with the increase of ion concentration. The critical temperature of the hydrogels could decrease to −48.4 °C with the addition of 30 wt% CaCl_2_ salts. Wu et al. [27] put forward a kind of carrageenan/polyacrylamide-based double-network hydrogels with high LiCl concentration and small amounts of KCl for low-temperature flexible supercapacitors. The obtained hydrogels could display strong tensile and conductive properties down to −40 °C. Ge et al. [25] utilized a one-step acrylamide polymerization in the presence of cellulose nanofibrils and LiCl to produce PAM/CNF double-network hydrogels. They found that the freezing tolerance of hydrogels could lower to −80 °C after introducing LiCl. Dai et al. [29] employed butanediol and N-hydroxyethyl acrylamide monomer with a multi-hydrogen bond structure to construct LiCl/p(HEA-co-BD) conductive hydrogels. Due to the hydration of LiCl and the interaction between the multi-hydrogen bond with H_2_O molecules, the LiCl/p(HEA-co-BD) hydrogels exhibited better anti-freezing performance with a lower freeze point of −85.6 °C in comparison with other hydrogels. The LiCl/p(HEA-co-BD) conductive hydrogels were used as the raw materials for a flexible sensor to monitor the compression and stretching signals. Diao et al. [30] synthesized a kind of double-network (DN) hydrogel by adding the chitosan-poly(acrylic acid-co-acrylamide) composite hydrogels into an Fe_2_ (SO_4_)_3_ solution. Better conductivity and anti-freezing performance (−20 °C) were found in the DN hydrogels due to the incorporation of inorganic salts.

Meanwhile, adding organic zwitterions into hydrogels could also improve the anti-freezing properties of hydrogels. Sui et al. [26] proposed to utilize betaine and proline to enhance the anti-freezing properties of NH_4_Cl-containing Ca-alginate/polyacrylamide hydrogels. The hydrogels were prepared by a one-pot solvent displacement method. The anions and cations of organic zwitterion could combine with water molecules, and the imine of proline could also interact with water, inhibiting the freezing process of water. The resulting hydrogels displayed excellent anti-freezing performance and ionic conductivity at −40 °C, which broadened the working temperature range of flexible electronics, as shown in Figure 2b. By performing a free-radical copolymerization of 1-vinyl-3-(carboxymethyl)-imidazole (zwitterionic IL) and acrylamide in KCl solution, Liu et al. [28] created a specific sort of zwitterionic poly(ionic liquid) hydrogels. In addition to having exceptional self-healing and stretch properties, these hydrogels also had very favorable low-temperature freezing resistance at −20 °C.

In conclusion, as one of the most popular approaches, the addition of soluble ions to solvents can increase the conductivity of hydrogels while also reducing their freezing point by inhibiting the formation of ice crystals. However, too many soluble ions may cause unfavorable mechanical properties in hydrogels, which will prevent them from being utilized in real-world applications. Ionic liquids or organic solvents can be used as a solute to create long-term stable hydrogels [16].

(2) Introducing organic solvents into hydrogels

**Figure 3:**
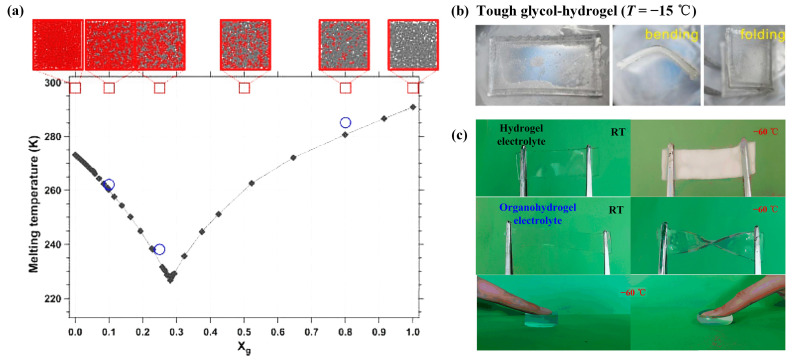
Effects of introducing organic solvents on the anti-freezing properties of hydrogels: (**a**) Melting temperature of aqueous glycerol changing with glycerol mol fraction; (**b**) Anti-freezing performance of cryoprotectant filled organohydrogels; (**c**) Images of hydrogels and organohydrogels electrolytes under low temperature. Reprinted with permission from [31,41,51].

Scholars have found that creating the binary solvent by introducing organic solvents into hydrogels could solve the freezing problem at low temperatures [50]. Strong hydrogen bonds form between organic molecules and H_2_O molecules, which compete with the hydrogen bonds between water molecules, preventing the formation of ice lattice at low temperature. Meanwhile, introducing organic molecules as part of the solvent of hydrogels can decrease the chemical potential of the liquid while maintaining that of a solid, resulting in a decrease of the hydrogel’s freezing temperature [51]. Figure 3a displays the melting temperature of aqueous glycerol, changing with glycerol mol fraction. It can be seen that adding a certain amount of glycerol into H_2_O can decrease the freezing temperature.

Hydrogels with the introduction of organic solvents are often named as organohydrogels. The typical organic solvents into hydrogels are ethylene glycol [13,14,32,52], glycerol [33,34,35,43,53,54], dimethyl sulfoxide [36,37,38], sorbitol [31,55] and polysaccharides, etc. Liu et al. [19] prepared a kind of photoresponsive metallopolymer organohydrogels by creating reversible crosslinks from photoresponsive Ru-thioether coordination bonds in polymer networks. The glycerol/water binary solvent, which could operate in cold settings at −20 °C, was used to dissolve the photoresponsive metallopolymer organohydrogels. Shi et al. [18] prepared hydrogen-bonded supramolecular organohydrogels by gelating poly(vinyl alcohol) in the glycerol/water binary solvent. It endowed the resulting organohydrogels with improved tensile strength, elongation and a remarkable freezing tolerance. The organohydrogels displayed a much lower crystallization temperature of −96 °C and still maintained a certain elasticity without completely freezing, even at −78.5 °C. Ma et al. [39] synthesized a kind of nanocomposite organohydrogels, with the hydrogel’s polymer network consisting of poly(vinyl alcohol), phenylboronic acid-grafted alginate and polyacrylamide in the ethylene glycol binary/water solvent containing a redox graphene oxide network. The organohydrogels demonstrated dependable self-healing, long-lasting wetness and a good temperature endurance of −40 °C. Yang et al. [34] produced a kind of multifunctional adhesive organohydrogels by the nonequilibrium polymerization of hydroxyethyl acrylamide monomers into the glycerol/water binary solvent. The organohydrogels could retain superior adhesive property at a working temperature of −30 °C. Adding tannic acid-carbon nanotubes to a poly(vinyl alcohol) matrix dispersed in glycerol-water, He et al. [35] created conductive hydrogels with good anti-freezing capabilities at −30 °C and a long-term moisturizing performance of 10 days. Wang et al. [37] developed a “dimethyl sulfoxide/water in salt”-based chitosan hydrogel electrolyte by employing chitosan as the substrate, DMSO and water as the solvents and high concentrations of LiTFSI as the salt, for all-solid-state supercapacitors. The introduction of organic co-solvent realized the excellent temperature stability of hydrogels from −20 °C to 70 °C. Ye et al. [38] fabricated a kind of ion-conducting organohydrogels with high ductility and high stress by sol-gel transition of cellulose nanofibrils and poly(vinyl alcohol) in the DMSO/H_2_O binary solvent. The oxygen atom in DMSO could form a strong interaction with water molecules. By adjusting the ratio of DMSO/H_2_O (7:3), the freezing point of organohydrogels could be as low as −70 °C. Cryoprotectants were used by Chen et al. [31] to make Ca-alginate/polyacrylamide organohydrogels that were soaked in glycerol, glycol, sorbitol or mixtures of these. From Figure 3b, the organohydrogels possessed high freezing and drying resistance, which could remain soft even at −70 °C. Lu and Chen [36] introduced dimethyl sulfoxide into MMT/PVA hydrogel electrolytes, which could lower the freezing point of hydrogels below −50 °C. The MMT/PVA hydrogels displayed outstanding anti-freezing and thermal-stable capabilities, which were useful for the realization of all-temperature flexible supercapacitors. Rong et al. [14] prepared anti-freezing organohydrogels by dissolving poly(vinyl alcohol) polymer powders and poly(3,4-ethylenedioxythiophene)/polystryrene sulfonate solution in the ethylene glycol/H_2_O binary solvent. At −55.0 °C, the organohydrogels demonstrated steady flexibility and strain sensitivity. In the meantime, the solvent molecules could interact with the PVA chains to establish hydrogen bonds and trigger PVA crystallization, considerably enhancing the mechanical properties of the organohydrogels. By using sodium methacrylate and [2-(methacryloyloxy)ethyl] trimethyl NH_4_Cl as monomers during radical polymerization in a glycerol/water-mixed solution, Yang et al. [33] created a type of conductive organohydrogels. By incorporating glycerol into hydrogels, the organohydrogels were able to endure −20 °C for 24 h without freezing. Su et al. [32] proposed a kind of fast self-healing and repeatably adhesive organohydrogel composed of polyacrylic acid, poly(vinyl alcohol), borax, ethylene glycol and water. The organohydrogels were translucent, stretchy (by over 800%) and conductive within the temperature range of −60 °C to 60 °C. They were also capable of resisting freezing below −90 °C.

As seen above, adding organic solvents to hydrogels may lower their freezing points by interacting with the water molecules in the hydrogels. The introduction of organic solvents had the advantages of easy synthesis. However, hydrogels used for flexible electronic devices are usually required to be conductive. Conductive fillings (carbon compounds, inorganic salts, metal nanoparticles, etc.) are currently used to add electrical conductivity to several hydrogels. Hydrogels’ ionic movement speed may be decreased by the addition of an organic solvent with a low dielectric coefficient, leading to hydrogels with low conductivity.

Many scholars have introduced soluble ions into organohydrogel solvents. Lu et al. [43] produced a kind of organohydrogels electrolyte from hydroxypropyl cellulose (HPC)/poly(vinyl alcohol) hydrogel with a glycerol/water solvent containing LiClO_4_ using the solvent displacement strategy. Glycerol and inorganic salts added to the hydrogel matrix could successfully prevent water from freezing at low temperatures. This kind of organohydrogel electrolyte could work under −40 °C with its superior ionic conductivity. Peng et al. [42] studied a kind of double-network organohydrogel by doping conductive ZnSO_4_ into poly(vinyl alcohol)-polyacrylamide double-network hydrogels into the EG/H_2_O solvent. The excellent anti-freezing capabilities of the organohydrogels allowed them to maintain mechanical flexibility and exhibit outstanding bending ability without breaking at −50 °C, demonstrating their enormous potential to be used for artificial skin and in health management. In order to increase the cellulose hydrogels’ resistance to freezing, Zhang et al. [40] included ionic chemicals (ZnCl_2_/CaCl_2_) into the networks of hydrogels. They indicated that the existence of inorganic salts might prevent the formation of ice crystals and glycerol could promote the anti-freezing properties of hydrogels, resulting in the hydrogels maintaining their toughness at −70 °C. Hou et al. [41] prepared a kind of organohydrogel incorporating LiCl in glycerol/water solution with poly(vinyl alcohol) networks. From Figure 3c, hydrogel electrolytes could turn into an ice-like solid that could be easily broken at −60 °C, while organohydrogel electrolytes displayed excellent mechanical and electrical properties at −60 °C. The organohydrogels possessed excellent low-temperature tolerance with the ability to bend reversibly when operating at temperatures down to −60 °C. Wang et al. [13] synthesized a type of ionic conductive hydrogel by incorporating lignin nanoparticle (LNP) and AlCl_3_ into PVA matrix in the EG/H_2_O binary solvent. With the adaptation of EG and H_2_O binary solvent, the ionic conductive hydrogels possessed excellent anti-freezing abilities at −62.6 °C.

(3) Introducing liquids as solvents to form ionogels

Ionic liquids are usually in a liquid state at room temperature, which consist entirely of anions and cations. They possess the properties of excellent electric conductivity and flow like water with little evaporation. Ionogels will be produced if all of the water molecules in the hydrogels are replaced by ionic liquids. In a strict sense, the obtained ionogels are no longer hydrogels; they are formed by introducing ionic liquids into the physical or chemical cross-linking networks of polymer matrixes. However, because of the similar mechanical properties and application areas between ionogels and hydrogels, ionogels are also mentioned in this paper.

Compared with hydrogels, ionogels usually show exceptional ionic conductivity and thermal stability. Two common methods are utilized to prepare ionogels. One method is to modify the ionic liquid into polymerizable monomers, and then the monomers are polymerized into gels. However, by using this method, the ionic liquid is limited in the network and unable to move, resulting in a substantial decrease in its electrical conductivity compared with pure ionic liquids. The other method is to prepare ionogels using solvent substitution. Firstly, hydrogels are prepared and immersed in the ionic liquids at room temperature. Then, ionogels are obtained by dehydrating the ionic liquids. Due to the nonvolatility of ionic liquids, ionic liquids are fixed in the gel network to form the ionogels [46].

**Figure 4:**
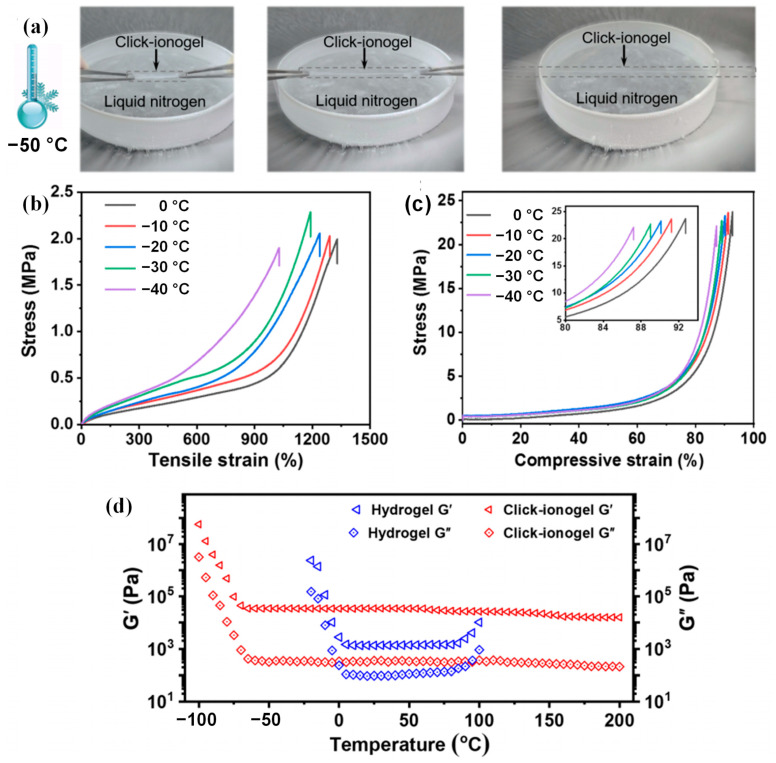
Mechanical properties of click-ionogels at low temperature: (**a**) Images of click ionogels stretching at approximately −50 °C; (**b**) Tensile stress-strain curves for click ionogels; (**c**) Compressive stress-strain curves for click ionogels; (**d**) Storage moduli (G′) and loss moduli (G″) of hydrogels and click-ionogels. Reprinted with permission from [45].

By using the N,N′-methylenebisacrylamide as a crosslinker, N,N-dimethylacrylamide as a monomer and 2,2-diethoxyacetophenone as a photo-initiator in an ionic liquid of 1-ethyl-3-methylimidazolium tetrafluoroborate, Yin et al. [20] produced a form of ionogels. The ionic liquid gave the ionogels exceptional electrical conductivity and anti-freezing ability while still exhibiting a respectable capacitance performance at −40 °C. By the photoinitiated polymerization of 2,2,2-trifluoroethyl acrylate and acrylamide in a hydrophobic 1-ethyl-3-methylimidazolium bis(trifluoromethanesulfonyl) imide-based ionic liquid, Xu et al. [44] generated physical crosslinked ionogels. The structure of fluorinated network endowed the ionogels with a remarkable anti-freezing resistance at −30 °C, which broadened the working temperature range of flexible electronic devices. By forming hydrogen bonds between poly(ethyl acrylate)-based elastomers and an ionic liquid based on 1-ethyl-3-methylimidazolium bis-(trifluoromethylsulfonyl)imide at room temperature, Cao et al. [21] created a type of high-performance ionogel. The ionogels exhibited excellent transparency, mechanical robustness and ultra-high stability. The ionogels retained their effectiveness in harsh conditions, such as in vacuum and moist environment from −70 °C to 100 °C, owing to the non-volatility and chemical stability of ionic liquids. Ding et al. [46] obtained a kind of transparent ionogel by locking the ionic liquid of 1-ethyl-3-methylimidazolium dicyanamide into charged poly(2-acrylamido-2-methyl-1-propanesulfonic acid)-based double networks. The ionogels presented both good mechanical strength and high ionic conductivity from −70 °C to 100 °C, which could be applied for flexible skin sensors. Ren et al. [45] synthesized click-ionogels with an ionic crosslinked network and a thiol-one click network. The sacrificial network was formed by ionic interactions between poly(1-butyl-3-vinyl imidazolium fluoborate) and benzene tetracarboxylic acid. The mechanical properties of click-ionogels at low temperatures were displayed in Figure 4. The ionogels exhibited high ionic conductivity, transparency, nonflammability and heat stability performance from −75 °C to 340 °C.

In summary, ionogels can provide excellent electrical conductivity over a wide range of temperatures. However, unlike inorganic salts, ionic liquids are hydrophobic and difficult to be added directly to hydrophilic gels. Therefore, the development of ionogels with low temperature resistance is still a great challenge.

(4) Modificating polymer network

Solutes may escape from the hydrogel network by simple physical mixing, which affects the anti-freezing performance of hydrogels. By altering the polymer network, solutes can be fixed to the hydrogel’s matrix to address this problem.

An innovative waterborne anionic polyurethane acrylate/polyacrylamide (EG-waPUA/PAM)-based dual-crosslinked hydrogel was created by Mo et al. [22]. The EG-waPUA precursor’s molecular structure was end-capped and had lots of end-double bonds. Even at −20 °C, this type of hydrogel, with its strong cooperative hydrogen bonding, displayed outstanding anti-freezing ability and long-term stability, which was beneficial for aqueous batteries to operate at sub-zero temperatures. By using free-radical polymerization, Pei et al. [23] created a kind of alkalified polyacrylic acid hydrogel, containing many carboxyl groups on the polymer chain. According to the experimental data, the alkalified PAA hydrogel with 10% KOH solution possessed a higher freezing point than the PVA hydrogel with the same amount of KOH, showing that the polarized terminal groups could further lower the freezing point of hydrogels.

In addition, learning from animals to utilize peptides to regulate the temperature of cells, peptides were added to hydrogels to endow them with a certain resistance at low temperature. Inspired by fish, Xu et al. [47] proposed a kind of novel anti-icing hydrogel by adding the anti-freeze protein of AFPS to the chemical crosslinking network copolymerized with acrylamide and 2-acrylamide-2-methylpropanesulfonic acid. The prevention of ice crystal development resulting from AFPS molecules enhanced the freezing resistant capability of the hydrogels down to −10 °C. Wiener et al. [48] were inspired by water trapping in freeze-proofing proteins, synthesizing supramolecular dimethylacrylamide and 2-(N-ethylperfluorooctane sulfonamide) ethyl acrylate copolymer hydrogels with water confined by the hydrophobic FOSA nanodomain crosslinks. The restriction of water in the hydrophobic FOSA nanodomains prevented water within the hydrogels from freezing, resulting in the fully hydrated hydrogels being stable down to −68 °C. Wang et al. [49] developed anti-freeze supramolecular hydrogels by using the copolymers of 2-hydroxyethyl acrylate and 2-(N-ethylperfluorooctane sulfonamide) ethyl methacrylate. The hydrogels could suppress the absorbed water from freezing down to −145 °C because of the nano-confinement of water within the hydrophobic parts. The excellent freeze-proofing ability should also be attributed to the hydrogen bonding of water to hydrophilic chains.

#### 2.1.2. Anti-Drying Properties

Hydrogels possess excellent properties due to the containing of water, but water in hydrogels can evaporate in an open environment, especially under high temperature. Eventually, hydrogels may become hard and brittle, lose mechanical flexibility and electrical conductivity, etc.

Just like the methods of improving the anti-freezing properties of hydrogels, adding soluble ions and alcohols into hydrogels can also improve the anti-drying properties of hydrogels. As for the addition of soluble ions into hydrogels, due to the colligative properties of salt solution, introducing high concentrations of soluble ions into hydrogels not only reduces the freezing point of the solution, but also reduces the surface vapor pressure of the solution and slows down the evaporation rate of the water, which improves the water-holding capacity of the hydrogels. Meanwhile, when the salt solution reaches a certain concentration, it absorbs water from the air. From the research of Ge et al. [25], adding LiCl into the PAM/CNF double-network hydrogels could effectively strengthen the interactions between water molecules in hydrogels, enhancing both the anti-freezing performance and anti-drying performance. When the salt concentration reached 50%, it could even absorb water from the environment, leading to a promotion in the quality of hydrogels.

**Figure 5:**
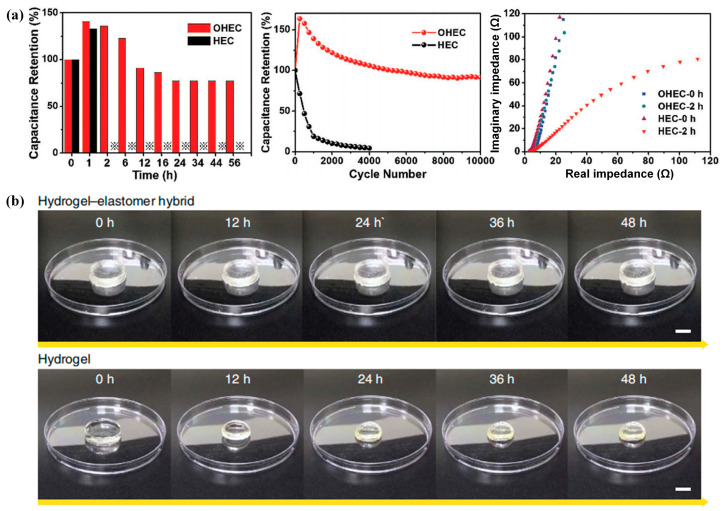
Anti-drying properties of hydrogels: (**a**) Electrochemical property of OHE supercapacitor at 80 °C; (**b**) Comparison of hydrogel-elastomer hybrid and hydrogel during the dehydration. Reprinted with permission from [58,62].

In addition, for the introduction of organic solvent into hydrogels, because of the abundant hydroxyl groups in organic solvents, the hydrogels’ ability to resist freezing can be enhanced by substituting a portion of water with organic solvents, such as ethylene glycol and glycerol, which also helps to prevent water from being dehydrated, resulting in the hydrogels maintaining regular properties under high temperature. Lou et al. [56] obtained a kind of tough double-network organohydrogel by soaking PAMPS/PAM hydrogels in the ethylene glycol solution containing LiCl. Due to the high boiling point of ethylene glycol, the organohydrogels could retain high flexibility and transparency under 120 °C. Han et al. [57] used the mixture of water and glycerol as solvents. The strong hydrogen bond between water and glycerol locked water firmly in the hydrogels’ network, resulting in excellent thermal stability. The hydrogels could be stored from −20 °C to 60 °C for a long time with fine properties. Li et al. [58] assembled a flexible supercapacitor by using organohydrogel-based electrolytes (OHEs) and an activated carbon-based electrode. The OHE was composed of PAMPS/PAM double-network hydrogels bathed in 4 M LiCl/ethylene glycol. It exhibited exceptional conductivity and outstanding anti-drying performance, which could maintain remarkable cycling stability. Compared with the hydrogels-based electrolyte capacitor, the organohydrogel-based electrolyte capacitor displayed excellent electrochemical property and interfacial compatibility at 80 °C, as shown in Figure 5a.

In addition, adding nanoparticles to hydrogels has increasingly gained popularity as a way to improve their properties in recent years. Several investigations have indicated that adding nanoparticles to polymer hydrogel can considerably increase the hydrogels’ durability to strength, temperatures and salinity [39,59,60,61]. One way to increase the bound water content in hydrogels is through the ability of nanoparticles to absorb water and hydrated ions via hydrogen bonding and dipole interaction. In addition, nanoparticles can create hydrogen bonds by functioning as physical crosslinkers with amide groups and other types of groups. In addition to increasing the network density of hydrogels, this physical crosslinking can significantly slow down the hydrolysis of amide groups, which enhances the durability and strength of the hydrogels provided by nanoparticles. Sheng et al. [60] investigated the effects of varying the concentrations of silica nanoparticles on the thermal properties of gel foams composed of a blend of fluorocarbon (FS-50) and hydrocarbon (APG0810) surfactants in a NaCl solution. By aggregating and establishing the network systems with surfactant in the liquid and plateau boundaries of bubbles, the added nanoparticles improved the thermal endurance of foam. The network structures could function reliably at temperatures as high as 65 °C, which reduced foam drainage and coarsening and improved the foam liquid film’s ability to withstand outside disturbances. Nano-SiO_2_, water-soluble phenolic resin and partially hydrolyzed polyacrylamide were utilized by Guo et al. [61] as the stabilizer, crosslinker and primary ingredient to create high-strength hydrogels. The results showed that the interaction between nano-SiO_2_ and the polymer might, to a certain extent, inhibit polymer degradation and increase the gel’s thermal stability up to 110 °C.

Moreover, as animal skin consists of an organic elastomer-like epidermis layer and a hydrogel-like dermis layer, the epidermis layer can inhibit the water evaporation from the dermis layer. Inspired by mammalian skins, for use in flexible electronics, tissue engineering, biomedical devices and other related fields, Yuk et al. [62] assembled tough hydrogels and widely utilized elastomers into hybrids, such as polydimethylsiloxane Sylgard 184, polyurethane, etc. From Figure 5b, the hydrogel-elastomer hybrids had less weight loss over 48 h in comparison with the uncoated hydrogels, which demonstrated the effective hydration-proofing of the hybrids. In order to crosslink the butyl rubber and create a strong stickiness between the butyl rubber and hydrogels, Floch et al. [63] developed a technique for coating a polyacrylamide hydrogel with a butyl rubber. Salt transfer in the aqueous solution and water dehydration were both reduced.

### 2.2. Application of Hydrogels Based on Thermal Stability in Electronic Devices

Nowadays, hydrogels have been extensively applied in flexible sensors, energy storage devices and other electronic devices.

#### 2.2.1. Application in Flexible Sensors

**Figure 6:**
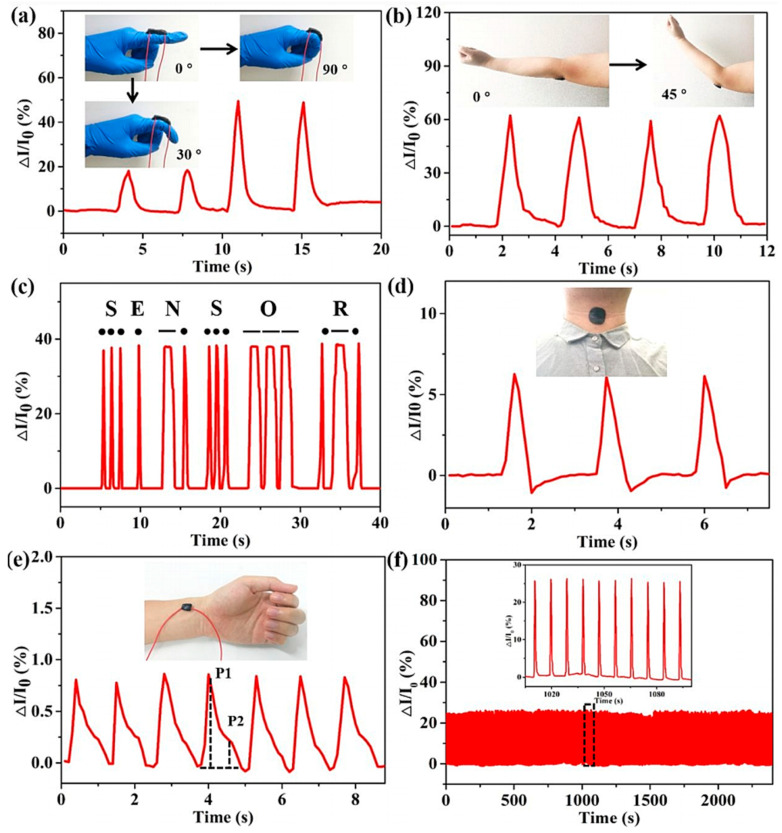
Detection of human motions by wearable organohydrogel-based sensors: (**a**) Sensor under finger bending with different angles; (**b**) Sensor under elbow bending with different angles; (**c**) Sensor by pressing; (**d**) Sensor under swallowing; (**e**) Sensor detecting pulse; (**f**) Durability measurement. Reprinted with permission from [39].

Flexible sensors are tools used to emulate the capabilities of sensory organs, which collect outside signals and transform them into different types of energy. According to the type of external signals, flexible sensors can be classified as temperature sensors, chemical sensors, stress/strain sensors, humidity sensors, etc. Current applications for hydrogel-based sensors include disease detection, pollution monitoring, artificial intelligence, field monitoring, etc. However, the stability of flexible sensors composed of hydrogels largely relies on the anti-freezing and anti-drying characteristics of hydrogels. With the volatilization of water under high temperatures or the freezing of water under low temperatures, the stability of hydrogel-based flexible sensors decreases. The methods mentioned above can improve the thermal stability of hydrogels. Diao et al. [30] studied strain sensors prepared based on chitosan-poly(acrylic acid-co-acrylamide) double-network hydrogels containing Fe_2_(SO_4_)_3_. Even at −20 °C, the strain sensors showed incredible sensitivity and dependability in monitoring the stretching and bending. The DN hydrogels, which were highly mechanical, conductive and freeze-resistant, offered a potential use in the area of wearable technology. Based on the TA-CNT-glycerol-PVA (TCGP) hydrogel, He et al. [35] produced a kind of strain sensors for the recognition of diverse human motions and electrodes for the recognition of electrophysiological information even in a rather extreme environment. With their superior anti-freezing, anti-dying and sensitive qualities, TCGP hydrogels hold great potential for use in bioelectrodes and wearable sensors. Ma et al. [39] prepared Alg-3-aminobenzeneboronic acid (PBA)/PVA/PAM polymer network organohydrogel in the rGO-contained EG/H_2_O binary solvent to fabricate the ultrasensitive wearable sensors. Figure 6 displayed the detection of various human motions by wearable organohydrogel-based sensors after being stored at −40 °C. Wearable sensors could be made from hydrogels with high temperature tolerance, long-time water retention and self-healing properties to precisely monitor human activity in harsh conditions. Peng et al. [42] employed PVA/PAM double-network organohydrogels containing ZnSO_4_ in the EG/H_2_O binary solvent as the materials of hydrogel-based flexible sensors for ionic skins. The organohydrogels displayed fast signal response time, excellent fatigue resistant features, outstanding freezing-proof property, remarkable flexibility and high sensitivity when the temperature was as low as −50 °C, which provided good potential applicability for artificial skin and health monitoring.

#### 2.2.2. Application in Energy Storage Devices

The development of effective energy storage technology is necessary for the rising global energy demand. Supercapacitors have gained a lot of attention due to their quicker charging and discharging rates and longer service lives. Solid electrolytes are an essential part of supercapacitor. Generally, the electrical conductivity of solid electrolytes is lower compared with that of liquid electrolytes, which limits the development of energy storage devices. The polymer hydrogel electrolyte possesses a relatively high electrical conductivity at room temperature, which attracts much attention in the field of supercapacitors. However, because of the existence of water, hydrogels unavoidably freeze below freezing point, reducing their electrical conductivity and mechanical capabilities. It can be challenging to improve the hydrogels’ conductivity while maintaining their flexibility at low temperature. Wu et al. [27] prepared anti-freezing CG/PAM DN hydrogels through the one-pot method as the materials of electrolytes for flexible supercapacitors. The flexible supercapacitor, composed of CG/PAM-7Li/K hydrogel electrolyte, exhibited outstanding electrochemical characteristics under −40 °C. Ge et al. [25] utilized PAM/CNF double-network hydrogels containing LiCl as the electrolyte for flexible supercapacitor. Because of the interaction between CNF and LiCl, the flexible supercapacitor with hydrogels electrolyte displayed high tensile strength and ionic conductivity at −40 °C, which proved to be a trustworthy electrochemical device for flexible electronics under extremely cold conditions. Hou et al. [41] adopted the organohydrogels electrolyte with LiCl in glycerol aqueous solvent and rGO films with microvoids as electrodes for the supercapacitor. The supercapacitor exhibited outstanding flexibility and temperature tolerance with the operating temperature down to −60 °C. By the seamless integration between EMIMBF4-based Bucky electrodes and an EMIMBF4-based ionogel electrolyte/separator, Yin et al. [20] produced a type of all-solid-state supercapacitor. It displayed excellent mechanical robustness and self-recoverability from −40 °C to 100 °C, which could be used as safe energy storage devices for flexible electronics. Through the random copolymerization of zwitterionic monomer and 2-hydroxyethyl acrylate with LiCl, Yang et al. [64] created zwitterionic polymer hydrogel electrolytes. The dissociation of LiCl might be enhanced by the cationic and anionic counterions on the polymer chains. The conductivity and anti-freezing capabilities of the electrolytes were significantly impacted by the salt content, resulting in the hydrogels displaying outstanding stretchability and conductivity at −40 °C.

Moreover, hydrogels can be used as conformal packaging. An anti-freezing, anti-drying and flexible network was fabricated by Shen et al. [65] using Ca-alginate/polyacrylamide tough organohydrogels with glycerol as the base. The organic hydrogels could endure temperatures as low as −50 °C while maintaining their 3D structures over a long period of time in dry or vacuum environments. They were used as a packaging material for both highly stretchable liquid metal conductors and inflexible electronic components. The stretchy, flexible equipment had the long-time stability needed for applications in demanding environments, as well as a high mechanical durability that could endure strains of up to 1000%.

## 3. Thermoresponsiveness of Hydrogels with Applications in Electronic Devices

According to the crosslinked type, physical and chemical crosslinked hydrogels are two categories of hydrogels. Non-reversible crosslinks are usually formed in the chemical crosslinked hydrogels. On the contrary, physical crosslinked hydrogels are generally crosslinked by dynamic noncovalent bonds, such as host-guest interaction, ionic interaction, ligand coordination and hydrogen-bonding interaction. They exhibit the characteristics of reversibility, repairability and responsiveness due to the non-covalent interactions. Most hydrogels are both physically and chemically crosslinked. Hydrogels with physical crosslink can sense and respond to the changes from the environment, including temperature [66,67,68], pH [66,68,69,70], electric field [69,71,72], magnetic field [73] and light [68,74,75,76]. Considering the importance of temperature in nature, temperature is the stimulus that has been investigated the most in the area of stimuli responsive polymers [77].

### 3.1. Thermoresponsiveness of Hydrogels with Influencing Factors

**Figure 7:**
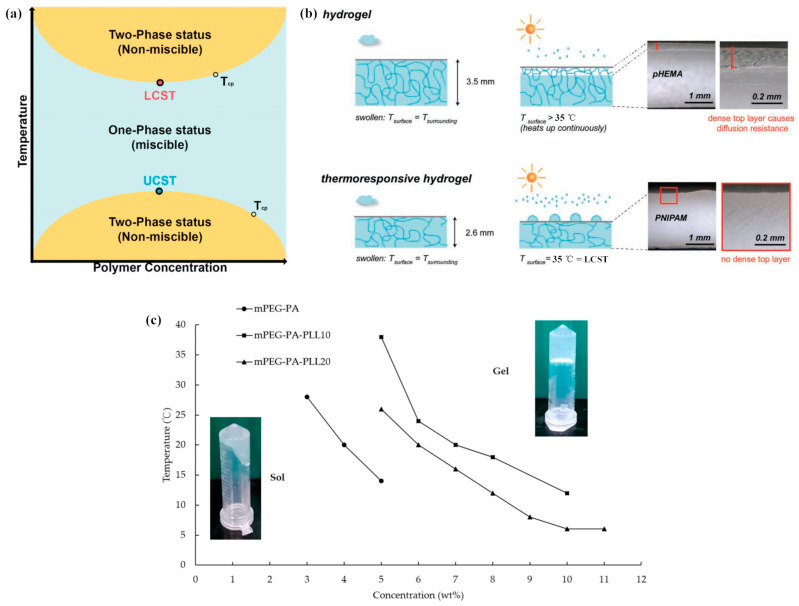
Thermoresponsiveness of hydrogels: (**a**) LCST and UCST of thermoresponsive polymers; (**b**) Thermal behavior of thermoresponsive PNIPAM hydrogels compared with conventional PHEMA hydrogels by heating; (**c**) Sol-gel transition of mPEG-PA and mPEG-PA-PLL with different PLL length. Reprinted with permission from [78,99,102].

Thermoresponsiveness refers to a critical change of parameters over a small temperature range, rather than a progressive thermosensitivity. Thermoresponsive hydrogels undergo discontinuous shape, appearance or size change with the change of temperature. Compared with conventional hydrogels, thermoresponsive hydrogels can experience a reversible sol-gel transition with the variation of temperature, which is attributed to non-covalent interactions, such as hydrogen bonding, van der Waals interaction, ion interaction, metal-ligand interaction, hydrophobic interaction and changes in entropy and enthalpy. Unlike the conventional hydrogels, amphiphilic polymers with both hydrophilic and hydrophobic components make up thermoresponsive hydrogels. They usually contain different hydrophobic groups (e.g., methyl, ethyl and propyl groups), leading to the variation of physical and conformational properties with temperature. The temperature when the hydrogels undergo a sol-gel transition is called the critical solution temperature. Lower critical solution temperature (LCST) polymers and upper critical solution temperature (UCST) type polymers are two kinds of thermoresponsive polymers based on their various interactions. When the temperature exceeds the UCST, the thermoresponsive polymers with UCST dissolve in the aqueous solution, whereas thermoresponsive polymers with LCST dissolve in the aqueous solution when the temperature is below the LCST [78], as shown in Figure 7a. Thermoresponsive hydrogels with UCST behaviors usually come from hydrogen bonding and ion interaction, which can be classified as hydrogen bonding and Coulomb interaction-based UCST polymers. Some typical polymers used in thermoresponsive hydrogels are displayed in Table 2.

For the utilization of thermoresponsive hydrogels, temperature is often used as a stimulus source to induce the volume change of hydrogels in response [97]. At the operating temperature above the critical solution temperature, thermoresponsive hydrogels with UCST absorb water to reach the swollen state, while they release water and shrink below the critical solution temperature. Comparatively, thermoresponsive hydrogels with LCST behave oppositely with those with UCST around the critical solution temperature. For example, poly(N-isopropylacrylamide) hydrogel is a typical thermoresponsive hydrogel, which can change from a hydrophilic state to a hydrophobic state around the LCST at approximately 32 °C [98]. The affinity between water and polymer has a significant impact on it. Hydrophilic amide groups in the side chains of PNIPAM interact with water molecules through hydrogen bonds when the temperature is lower than LCST. Hydrogen bonds between copolymer and water break when the operating temperature exceeds the LCST, causing water molecules to rapidly diffuse throughout the bulk phase. Hence, the hydrogel collapse is caused by hydrophobic interactions between isopropyl groups inside the polymeric network. Due to the development of hydrophobic areas in reaction to heating, PNIPAM hydrogel changes from a swollen to a collapsed condition. As shown in Figure 7b, conventional hydrogels such as poly-hydroxyethyl methacrylate (PHEMA) hydrogel form a dense top layer with high diffusion resistance, which reduces the cooling rate and results in a higher hydrogel surface temperature [99]. Compared with PHEMA hydrogel, due to its rapid evaporation when heated, no dense top layer formed in thermoresponsive PNIPAM hydrogel and the temperature was close to LCST. However, rapid water release may cause the polymer chains of PNIPAM to contract or collapse abruptly and the phase separation of PNIPAM hydrogel system occurs, when the temperature is above LCST [100]. And PNIPAM recovers slowly after shrinkage. Zhang et al. [101] utilized poly(ethylene glycol) as the pore-forming agent to synthesize macroporous thermoresponsive PNIPAM hydrogels. The structure of hydrogels prompted the entrance and exitance of water and heat into the hydrogels matrix at the temperature above LCST, resulting in an excellent response of hydrogels to the external temperature changes during deswelling and reswelling. Cheng et al. [66] added alkali into PNIPAM layer to accelerate the recovery rate of PNIPAM layer bending caused by high temperature. Zhao et al. [68] utilized poly(dimethylaminoethyl methacrylate) (PDMAEMA) to produce thermoresponsive hydrogels with good recoverable performance.

The critical solution temperature value and volume ratio during expansion and contraction of hydrogels are vital factors to evaluate the thermoresponsive performance of hydrogels. They are usually affected by composition and molecular weight of polymers or copolymers, concentration of monomers, and crosslinking content, degree and method. First of all, the composition of polymers or copolymers can affect the critical solution temperature value and volume ratio during expansion and contraction of hydrogels. Sponchioni et al. [96] proposed a kind of biodegradable zwitterionic nanoparticles with poly(sulfobetaine-co-sulfobetaine) (p(SB-co-ZB)) copolymers. The polymeric nanoparticles displayed thermoresponsive characteristics. The UCST value changed with the variation of length and composition of p(SB-co-ZB) copolymers. Št’astná et al. [77] examined the sensitivity of single network (SN) and double-network hydrogels composed of poly(N,N′-diethylacrylamide) and polyacrylamide. The addition of PAM and the creation of double networks were found to have a considerable impact on the sensitivity of DN hydrogels. The PDEAM-based SN hydrogels displayed remarkable temperature and salt sensitivity. The deswelling of DN hydrogels was less intense and gradual than that of SN hydrogels as a result of temperature and salt-induced alterations because of the hydrophilic PAM groups. In addition, the critical solution temperature value of hydrogels changes with the molecular weight of polymers. Zhang et al. [92] synthesized a series of PiGMA samples with different repeat unit numbers. They found that the UCST depended on the solution pH values and PiGMA molecular weights obviously. The UCST value could increase from 23.2 °C to 93.2 °C with the increase of repeat unit number of polymer from 15 to 140. Cheng et al. [102] synthesized a triblock copolymer of methoxy poly(ethylene glycol)-poly(L-alanine)-poly(L-lysine) (mPEG-PA-PLL) and applied it to a hydrogel system. The results from Figure 7c demonstrated that mPEG-PA-PLL gelated at a higher temperature compared to mPEG-PA. Nevertheless, due to its massive molecular weight, the mPEG-PA-PLL’s gelation temperature dramatically dropped at the same concentration when the L-lysine repeating unit increased. Meanwhile, the concentration of monomers plays a crucial role in changing the critical solution temperature value and thermoresponsive performance of hydrogels. Boustta and Vert [103] synthesized thermo-responsive hydrogels composed of N-acryloyl glycinamide and N-acryloyl L-alaninamide monomers. The latter was chosen in an effort to include hydrophobicity, chirality and UCST-based thermoresponsiveness. The gel-to-sol transition temperature, gel inconsistency and risperidone release rate were all observed to rise with higher N-acryloyl-alaninamide concentration. Utilizing 2-methyl-2-propenoic acid-2-(2-methoxy ethoxy) ethyl ester and oligoethylene glycol methyl ether methacrylate (OEGMA) as thermosensitive monomers, Liu et al. [104] produced thermoresponsive microgels. Their LCSTs and particle sizes were investigated. Higher OEGMA content brings a higher LCST of the obtained microgels. With the decrease of surfactant sodium dodecyl sulfate dosage, the particle sizes of obtained microgels increased.

In addition, the crosslinking content, degree and method of copolymers can also impact the critical solution temperature value and thermoresponsive behavior of hydrogels. Schlattmann and Schönhoff [105] studied the influence of crosslinker content on the phase transition of thermoresponsive poly(N-isopropylacrylamide)/N,N′-methylenebisacrylamide microgels. The crosslinker content increased the volume phase transition temperature while also significantly lowering the transition enthalpy. Nun et al. [106] synthesized a PNIPAM core-shell hydrogel by controlling the growth process of PNIPAM shell together with the crosslinker methylene-bis-acrylamide on the silica surface. They indicated that the thermoresponsive behavior (such as LCST temperature, LCST and UCST-like behavior) of hydrogels could be tuned by changing the crosslinker addition way. Xia et al. [107] synthesized nano-structured thermoresponsive PNIPAM hydrogels through building a nano-structured architecture using activated nanogels as nano-crosslinkers. The hydrogels displayed a high thermoresponsive swelling ratio of 8000% as temperature declined from 45 °C to 15 °C and a short shrinking time of 6 min from completely swollen to completely collapsed after heating from 25 °C to 55 °C. Chen et al. [108] proposed a kind of shape memory hydrogel, utilizing the combination of both strong and weak hydrogen bonding. Strong H-bonding was formed between poly(vinyl alcohol) and tannic acid (TA) functioning as the “permanent” crosslinkers and weaker H-bonding between PVA chains as the “temporary” crosslinkers. Remarkable temperature-responsive shape memory was given to the PVA-TA hydrogels by the reversible breakdown and formation of weak hydrogen bonding, leading to the hydrogels recovering to their original shapes in a few minutes. Imran et al. [109] utilized hydrophilic polyrotaxane as a movable crosslinker and N-isopropylacrylamide (NIPAM) as a monomer to produce thermoresponsive hydrogels. The hydrogels displayed a higher swelling capacity and faster thermoresponsive behavior compared with typical PNIPAM hydrogels due to the hydrophilicity and movability of the crosslinker, which could shrink full in about 10 min compared with N,N′-methylenebisacrylamide for more than one day. Sun et al. [110] produced a kind of nanomicelle thermoresponsive hydrogel using self-assembled PEO-PPO-PEO micelles with vinyl functional groups and served as multifunctional crosslinking sites for polymerization of AAM monomers. Without using any chemical crosslinkers, the hydrogels exhibited exceptional tensile and compressive capabilities. Ling et al. [93] studied a thermosensitive poly (acrylamide-co-acrylonitrile) hydrogel with UCST behavior. The findings demonstrated that hydrogel with various UCST temperatures could be produced by varying the degree of AAM dehydration. With the 101% grafting yield of AAM and 23% dehydration, the hydrogel surface showed optimal performance at approximately 32 °C. Li et al. [111] utilized dynamic acylhydrazine bonding and micellization crosslinking to create a new hydrogel (HA-az-F127 hydrogel) based on hyaluronic acid and biocompatible Pluronic F127. At room temperature, F127 was self-assembled into a micellar solution. The hyaluronic acid added with hydrazine and the F127, added with benzaldehyde to make micellization, produced the acylhydrazine bonds. Quick gelation and shear thinning were found in the hydrogels with dynamic covalent chemically and micellar physically double-crosslinked networks, which could be applicable in promoting burn wound healing. Li et al. [112] fabricated a remoldable hydrogel through the combination of dual reversible crosslinking with various relaxation time scales. Chemical crosslinking was formed by Schiff-base bonding between the surface-primary amine-rich silica nanodots and benzaldehyde-terminated F127 (PEO-PPO-PEO) triblock copolymers below 10 °C. Physical crosslinking was formed between the hydrophobic PPO segments when the temperature increased beyond 15 °C. The latter network was weak and showed a quick relaxation while the first one showed a longer relaxation time. The hydrogel’s distinctive structural properties gave it a high degree of stretchability and self-healing, which made it useful for capturing fingerprint data for identification purposes. Using dynamic chemical oxime bonding between alkoxyamine-terminated Pluronic F127 (AOP127) and oxidized hyaluronic acid, and also using the hydrophobic association of AOP127, Li et al. [113] produced a dual dynamically crosslinked hydrogel for a physical postoperative anti-adhesion barrier. The hydrogel exhibited high temperature sensitivity, which demonstrated a significantly higher modulus and better stability compared with the Pluronic F127 hydrogel at 37 °C.

### 3.2. Application of Hydrogels Based on Thermoresponsiveness in Electronic Devices

Thermoresponsive hydrogels with discontinuous volume change usually possess the virtues of intelligent response to temperature, superior biocompatibility, unique water absorption and retention and reversibility, which have been used to fabricate all kinds of sensors, actuators, supercapacitors, biomimetic skin for intelligent wear, medical treatment, energy storage and conversion and electronic cooling.

#### 3.2.1. Application in Sensors

**Figure 8:**
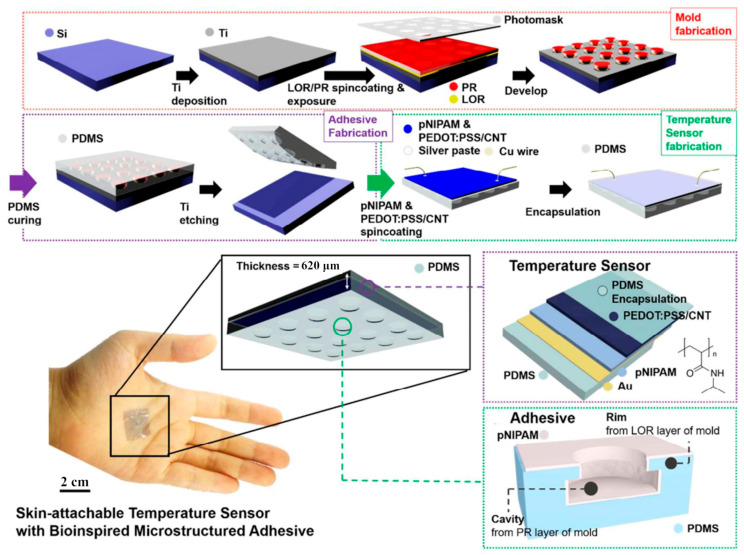
Fabrication process and the schematic illustration of PNIPAM hydrogel-based temperature sensor with the octopus-mimicking microstructured adhesive. Reprinted with permission from [114].

Numerous studies have been conducted to build sensors from thermoresponsive hydrogels. The sensing mechanism is generally based on converting the temperature signals to volumetric or electrical responses. Under the temperature effect, the expansion and contraction of hydrogel fibers can be translated into capacitance changes between electrodes, and the migration of ions varies with temperature. Because hydrogels’ conductivity and ion mobility are correlated, their resistance is temperature-sensitive and can be used to produce temperature sensors. Oh et al. [114] coated PNIPAM hydrogels on the resistor-type temperature sensor for medical and healthcare monitoring. The fabrication process and the schematic illustration of the temperature sensor are shown in Figure 8. The PNIPAM hydrogel-based temperature sensor exhibited a high thermoresponsive capacity of 2.6%·°C^−1^ between 25 and 40 °C. By incorporating thermoresponsive N-isopropylacrylamide into another conductive double-network hydrogel made of poly(vinyl alcohol)-graphene oxide and polyacrylic acid-Fe^3+^, Feng et al. [115] created a functional ionic hydrogel-based flexible sensor. The addition of a thermoresponsive network made it possible for the sensor to properly record changes in body temperature, enabling applications such as individualized health monitoring and human–machine interfaces. Meanwhile, the thermal recognition of a target analyte can induce a volumetric change of hydrogels, which can be utilized for sensors. Leu et al. [116] manufactured low-cost point-of-use hydrogel-based microfluidic sensors with short response times. Arrays of smart hydrogel pillars inside sub-millimeter channels were located upon microfluidic devices. When these pillars contacted aqueous solutions containing a target analyte, they swelled or shrank, thereby changing the microfluidic channel resistance to ionic current flow. Hence, resistance measurements could be used to transduce hydrogel swelling changes into electrical signals.

#### 3.2.2. Application in Actuators

**Figure 9:**
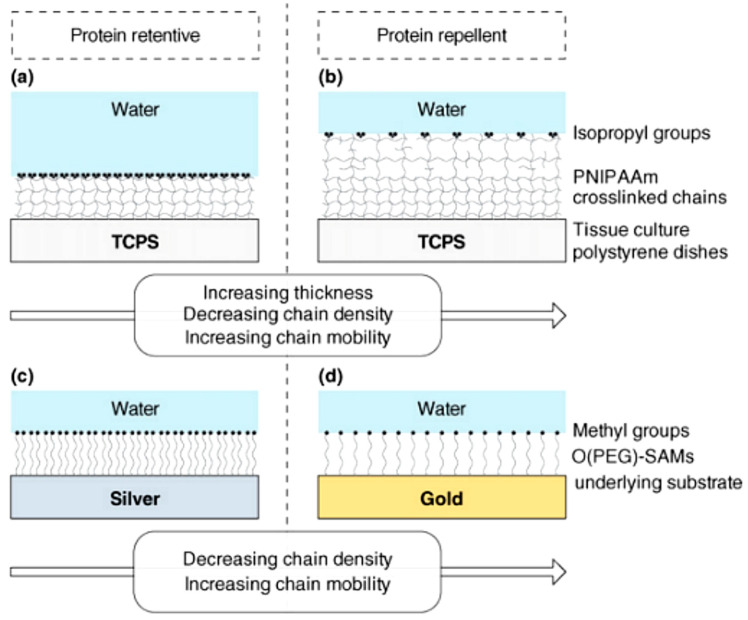
Hydrodynamic diameter variation of crosslinked PAM/PNIPAM due to temperature. (**a**) Schematic plot of e-beam-grafted insoluble PNIPAM surface; (**b**) PNIPAM chain density decreases with the increase of graft thickness; (**c**) Schematic plot of methoxy-terminated oligo self-assembled monolayers on silver; (**d**) Less compacted self-assembled monolayers forming on gold. Reprinted with permission from [120].

In order to achieve the reversible motions and volume/shape flexibility required for actuator operation, stimuli responsive hydrogels are thought to be appropriate for the manufacturing of actuators. Thermoresponsive actuators can convert the heat to mechanical energy to generate force and motion by using the deformation of the material itself, and have been widely applied in diverse areas such as biosensing systems, targeted drug delivery, substances separation and extraction, regenerative medicine, microfluidic valves and controllers and related fields. Since the accurate transport and release of drugs are the key points in medical drug therapy, intelligent drugs with directional release have been proposed by adopting thermoresponsive hydrogel delivery systems. The hydrogels can be used as carriers to coat and carry drugs and deliver drugs to the designated lesion site by controlling the hydrogel’s temperature. When the drugs reach the lesion site, the carrier is opened to release the drugs, which can realize directional transport and release of drugs. Cirillo et al. [117] proposed to utilize thermoresponsive hydrogel films as the carriers of non-steroidal anti-inflammatory drugs. N-isopropylacrylamide and N,N′-ethylenebisacrylamide were chosen as monomers and crosslinkers for thermoresponsive hydrogels, respectively. The hydrogel film’s temperature-dependent swelling or deswelling and the therapeutics diffusion across the network of the polymer both contributed to the drug release. Due to the significant extrusion phenomenon determined by the hydrophobic contacts between the isopropyl groups of NIPAM moieties, higher releases were seen while the temperature was above the LCST. In case of neurological oxidative stress, Dong et al. [118] put forward the use of an injectable thermoresponsive chitosan/gelatin/Glycerol phosphate (C/G/GP) hydrogel to modulate the release of the phenolic antioxidant ferulic acid. Under the gelation temperature of 32.6 °C and gelation period of 75.58 s, the C/G/GP hydrogel could guarantee an outstanding clinical applicability. Lv et al. [119] prepared crosslinked polyacrylamide soft hydrogel nanoparticles grafted with poly(N-isopropylacrylamide) chains. Affected by the shrinkage or inflation of PNIPAM chains with temperature, the size of the hairy particles changed accordingly, which could be used for controlled drug delivery and other biomaterial areas. Meanwhile, thermoresponsive hydrogels could be used as carriers for cells’ directional transfer. Silva et al. [120] reviewed the smart thermoresponsive coatings and surfaces for tissue engineering. Thin layers of crosslinked poly(N-isopropylacrylamide) were very attractive to act as the molecular switch for cell adhesion and detachment. From Figure 9, when the temperature was above LCST, hydrogels could be used as the substrate for cell growth and adhering to the surface of hydrogels. On the contrary, when the temperature was below LCST, the surface characteristics of hydrogels changed, resulting in the grown cell sheets being separated without biological enzymes. The bioengineered human corneal endothelium was created in vitro by Lai et al. [121] through the temperature-modulated detachment of cultivated cell sheets from thermoresponsive hydrogel substrates. The thermoresponsive poly(N-isopropylacrylamide)-grafted surface was used to cultivate the bioengineered human corneal endothelial cells for three weeks at a temperature of 37 °C before reducing the temperature to 20 °C to trigger the separation of the cells as a laminated sheet.

#### 3.2.3. Application in Microfluidic Valves and Controllers

**Figure 10:**
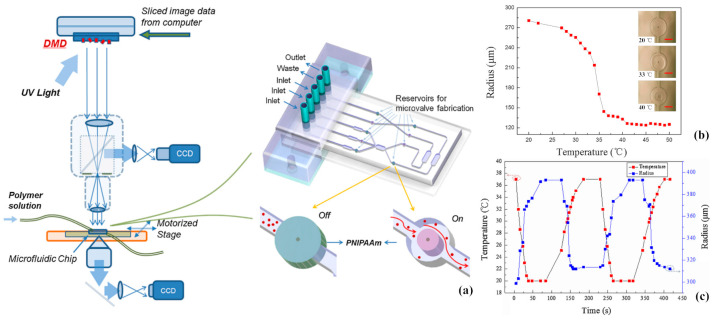
Microfluidic chip integrated with PNIPAM microvalves: (**a**) Schematic drawing of the PNIPAM microvalve; (**b**) Radius of PNIPAM microvalve with temperature; (**c**) Temperature cycling test results of PNIPAM microvalve with temperature. Reprinted with permission from [122].

Thermoresponsive hydrogels show great potential to be used as materials for microfluidic valves and controllers, due to their special environmental response characteristics and gradual controllable swelling rate. Huang et al. [122] fabricated an in-channel thermoresponsive PNIPAM microvalve on a microfluidic chip by employing UV light-based optical maskless exposure technology. Figure 10 shows the design and thermal performance of the microfluidic chip integrated with PNIPAM microvalves. It could be seen that by utilizing the discontinuous volume phase transition property of PNIPAM hydrogels, the PNIPAM microvalve could be iteratively opened and closed by adjusting the temperature between 20 °C and 37 °C. When the PNIPAM microvalve was at room temperature, it swelled and blocked the microchannel; whereas, it shrank and opened the microchannel when the PNIPAM microvalve was at a relatively high temperature above LCST. Wang et al. [123] fabricated a thermally actuated, self-regulated poly(N-isopropylacrylamide) hydrogel valve for flow control in pneumatically driven. Taking the advantage of the reversible volumetric change of hydrogel with temperature, the valve could realize flow control with the virtues of no moving parts, minimal dead volume and little leakage while being contaminant free. Tudor et al. [124] synthesized thermoresponsive crosslinked tributylhexyl phosphonium sulfopropylacrylate (PSPA) hydrogels as a temperature controller for microfluidic devices. The hydrogels could shrink by 56% when the temperature increased from 20 °C to 70 °C, and returned to their original size when the temperature returned to 20 °C. The hydrogels could shrink and reswell repeatedly under the temperature cycling ranging from 20 °C to 50 °C, which resulted in a flow rate of microfluidic channel changing from 27 nL·min^−1^ to 110 nL·min^−1^. Mishra et al. [125] designed fluidic elastomer actuators with micropores that could expand and contract autonomously in response to temperature for autonomic thermoregulation, by utilizing two kinds of hydrogel materials. Polyacrylamide-based thermally expanding hydrogels were used as the dermal layer for pore diameters expanding with temperature. Poly-N-isopropylacrylamide-based thermally contracting hydrogels were used as the strain-limiting actuator body for pore contraction, which could restrict fluid loss and restore operation. By utilizing this structure, cooling rates could improve by more than 200%.

#### 3.2.4. Application in Flexible Energy Storage Devices

**Figure 11:**
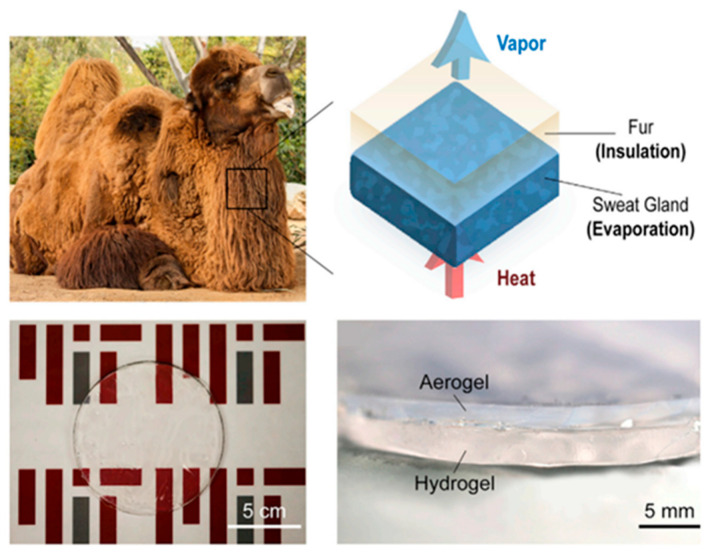
Concept of hydrogel-aerogel cooling. Reprinted with permission from [128].

Since hydrogels contain a large amount of water, this allows them to have thermal and conductive properties analogous to liquids, while maintaining the dimensional stability of solids, which is ideal for flexible energy storage devices. Zhou et al. [126] developed a thermoresponsive smart window with excellent thermal energy storage performance by dispersing poly (N-isopropylacrylamide) hydrogels particles in water to form water-rich thermoresponsive liquid within glasses. The thermoresponsive hydrogel-based structure acted as an energy storage layer with an additional function to absorb and store energy for smart window. When the temperature was below the LCST, water molecules within the hydrogels enhanced the solar transmission rate of windows to heat the room, while water molecules were released from the hydrogels above LCST, resulting in shrinkage particles causing light scattering. The structure could reduce 44.6% energy consumption compared with the traditional glass due to the hydrogel-derived liquid within the glasses. Yin et al. [127] produced a kind of room-temperature phase change organohydrogel (PCOH) consisting of disodium phosphate dodecahydrate (DPDH) as phase-change hydrated salts and polyacrylamide glycerol hydrogels through a facile photoinitiated one-step in situ polymerization process. Comprising environmentally friendly DPDH hydrated salts PCMs into anti-drying 3D networks of PAM organohydrogels could break through the solid rigidity and melting leakage. The PCOHs demonstrated excellent phase-change temperature control and energy storage capabilities along with outstanding anti-drying, flexibility, form stability and thermal cycle stability. Inspired by the fur layer of desert animals, Lu et al. [128] presented an evaporation-insulation cooling concept based on hydrogels and aerogels, as shown in Figure 11. Aerogels were composed of hydrophobic silica with a porosity of 95% and a thermal conductivity half that of air. The double layer structure relied on water evaporation from hydrogels through highly porous aerogels with ultra-low thermal conductivity and minimized heating from the surrounding, which could prolong the lifetime of cooling package by 400% in contrast to the traditional single layer design. The findings provided design criteria for evaporation-insulation systems.

#### 3.2.5. Application in Thermal Dissipation of Electronics

**Figure 12:**
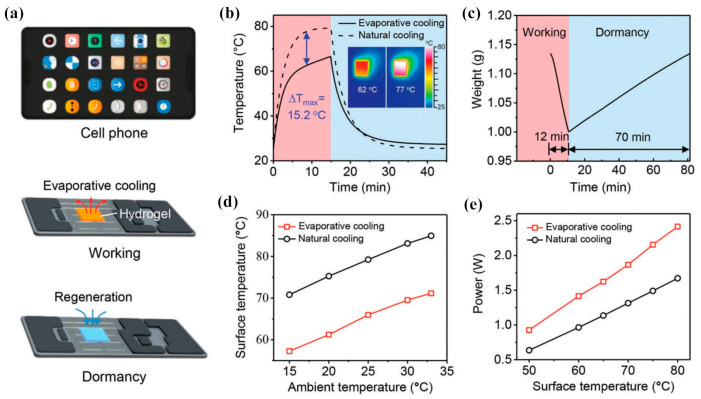
Evaporative cooling capability of Li-PAM hydrogel for mobile phone chip: (**a**) Cooling principle of Li-PAM based hydrogel; (**b**) Temperature variations of chips with and without the hydrogel; (**c**) Weight variation of the hydrogel during the cooling and regeneration processes; (**d**) Chip temperature variation with ambient temperature during heating; (**e**) Maximum allowed power for chip with and without the hydrogel. Reprinted with permission from [129].

Thermoresponsive hydrogels are sensitive to temperature, exhibiting excellent properties similar to spontaneous sweat, which is beneficial for the thermal dissipation of electronics. Pu et al. [129] proposed a self-adaptive evaporative cooling technology by utilizing a lithium- and bromine-enriched polyacrylamide hydrogel to improve the energy efficiency of electronic devices, as shown in Figure 12. Water inside the hydrogels could vaporize to remove the waste heat quickly and harvest water molecules from the environment to regenerate itself. It can be seen in Figure 12b that the temperature of a simulated phone chip was reduced by 15.2 °C under 2229 W·m^−2^ by utilizing the Li-PAM hydrogel. The hydrogel could lower the operating temperature of electronic devices by almost 17 °C, enhance the efficiency by 1% and increase the maximum power of the simulated chip by 45%. Huang et al. [130] utilized a thin layer of temperature responsive PNIPAM hydrogel as the biomimetic skin for the thermal management of handheld microelectronic devices. They obtained that the hydrogel could increase the cooling capability of a handheld device up to 4.9 times higher than the traditional passive cooling method. Pu et al. [131] used the temperature-responsive PNIPAM hydrogel as the sweating skin to dissipate electronic devices under 1555.5 W·m^−2^. They found that the heat transfer coefficient of hydrogel-based electronics was more than twice that of electronics using purely natural convection and radiation. The water content and swelling properties of hydrogels were sustained after 450 h of high-temperature and high-humidity aging tests and 60 cycles of fatigue tests. In addition, a heat sink with channels can be embedded with hydrogels. The flow and heat transfer of the heat sink can be controlled by spontaneously changing the channels’ cross-section area due to the volume change of thermoresponsive hydrogel. It is beneficial to the adaptive rapid cooling of high heat flux chips with local hotspots. The heat sink embedding with hydrogels can make uniform the temperature distribution of the heat source and improve the heat flux density of hotspots with low pump power consumption, which is helpful for the heat transfer enhancement of electronics. Zamengo and Morikawa [132] proposed utilizing polyvinyl alcohol hydrogels as the material of a heat sink for thermal management, indicating that water evaporation inside the hydrogel was dedicated for at least 51% total cooling power of the heat sink. Hydrogels could be chosen as useful materials for thermal management.

## 4. Thermal Conductivity of Hydrogels with Applications in Electronic Devices

Hydrogels have been utilized in various fields for all kinds of devices, especially for flexible electronics, due to their excellent properties in softness, mechanical robustness and biocompatibility. The thermal characteristics of hydrogels can greatly affect the stability of flexible electronic devices. The electronic devices embedded with hydrogels under a high heat flux can generate great heat in a short time, which causes rapid temperature rise around the circuit components, thus affecting the performance and service life of devices. Therefore, it is significant to investigate the heat transfer characteristics of flexible hydrogels.

**Figure 13:**
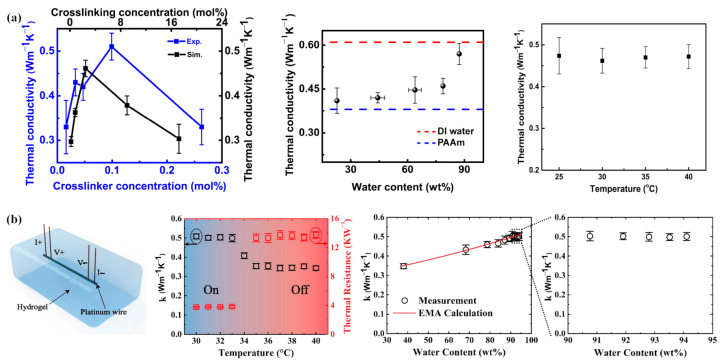
Thermal conductivity of hydrogels for electronic devices applications: (**a**) Thermal conductivity of PAM hydrogels; (**b**) Thermal conductivity of thermal switch based on PNIPAM hydrogels. Reprinted with permission from [133,137].

As thermal dissipation has been a great concern for electronics, the research of thermal conductivity for hydrogels in devices has attracted much attention from scholars. Tang et al. [133] studied the thermal conductivity of polyacrylamide hydrogels. From Figure 13a, the thermal conductivity of hydrogels varied with crosslinking density from 0.33 W·m^−1^·K^−1^ to 0.51 W·m^−1^·K^−1^ and enhanced by 40% with the water content changing from 23 wt% to 88 wt%. In addition, the hydrogels were insensitive to temperature ranging from 25 °C to 40 °C. Jiang et al. [134] fabricated a stretchable and self-healable thermal interface material by incorporating boron nitride nanosheets (BNNSs) into dual cross-linked polyacrylic acid hydrogels. They found that BNNS fraction and water content could apparently affect the thermal conductivity of BNNS/PAA hydrogel nanocomposites. A maximum value of 3.5 W·m^−1^·K^−1^ was realized at 50 wt% BNNS loading fraction and 40 wt% water fraction, which was four times that of pure hydrogel with 40 wt% water fraction. The dual-cross linked BNNS/PAA hydrogel-based nanocomposite exhibited high flexibility, self-healing ability and thermal conductivity, which could be utilized as thermal interface material (TIM) for electronic devices. This kind of soft gel TIM possessed the advantages of both solid TIMs and liquid TIMs, which was stable, non-perishable, non-flowable and had a tight stick to the devices for a better thermal conduction. Zhang et al. [135] prepared a kind of nanocomposite double-network (NCDN) hydrogel by introducing MXene nanomaterials into a hydrophobic associated polyacrylamide (HAPAM) hydrogel network and poly(N-isopropylacrylamide) hydrogel network. NCDN hydrogels presented more excellent thermal conductivity of 0.45 W·m^−1^·K^−1^ compared with other hydrogels, due to the addition of MXenes with its high thermal conductivity. Meanwhile, nanodiamond hydrogels could offer platforms for the controlled functionalization and delivery of a broad spectrum of therapeutic elements or biocompatible and biofunctional multilayer nanofilms. Usoltseva et al. [136] measured the thermal conductivity of concentrated nanodiamond aqueous solutions, covering the sol-gel transition state. The maximum thermal conductivity of nanodiamond gels was 0.69 W·m^−1^·K^−1^.

Moreover, some scholars have utilized the thermal conductivity variation of thermoresponsive hydrogels to produce diversiform flexible electronic devices, such as a thermal switch. Feng et al. [137] designed a thermal switch based on poly(N-isopropylacrylamide) hydrogels, as shown in Figure 13b. The thermal conductivity of the thermal switch could drop from 0.51 to 0.35 W·m^−1^·K^−1^ around the LCST of hydrogel, due to the transformation of hydrogels from being hydrophilic to hydrophobic caused by the temperature.

## 5. Conclusions and Outlooks

Using hydrogels as the materials of electronic devices is an important direction for the development of future flexible electronics. As hydrogels are sensitive to temperature, much attention should be paid to understanding and enhancing the thermal properties of hydrogels. In this review, the research of hydrogels thermal properties from scholars is reviewed and summarized. Thermal stability under extreme circumstances, thermal responsiveness and the thermal conductivity of hydrogels with their applications in electronics are discussed in detail. Hydrogels with high thermal performance can be adaptable to meet different needs for flexible electronics. Methods such as introducing soluble ions and organic solvents into hydrogels, forming ionogels, modifying polymer chains and incorporating nanomaterials can enlarge the working temperature range of hydrogels with high thermal stability. In addition, thermoresponsive hydrogels, with the virtue of intelligent response to temperature, can be applied widely to sensors, actuators, supercapacitors and biomimetic skin for electronics. Moreover, considering the higher requirement of thermal dissipation, the thermal conductivity of hydrogels is a crucial factor for their application in electronic devices.

Further studies are still needed on the thermal properties of hydrogels for electronic device applications. (1) In addition to improving the thermal stability, how to maintain the excellent mechanical, electrochemical and electrical properties of hydrogels with low cost and easy preparation is still challenging. (2) It is worth studying to incorporate other stimuli, such as pH, electric field, magnetic field and light into thermoresponsive hydrogels to expand the application of thermoresponsive hydrogels in all kinds of fields. (3) Although methods such as adding nanocomposites into hydrogels can improve the thermal conductivity of hydrogels, the enhancement effect is limited. Adopting other ways to improve the thermal conductivity of hydrogels further is required for electronic devices. (4) Combining phase-change materials with hydrogels to obtain high performance thermal interface materials is a promising way to achieve efficient thermal management. (5) It is necessary to fully explore the potential of hydrogel with high thermal performance and expand its application prospects in seawater desalination, thermoelectric power generation, medical treatment and in other fields. More attention should be paid to coupling hydrogels with other components or equipment to improve the comprehensive performance of the whole devices or systems.

## Figures and Tables

**Table 1:** General methods to enhance the anti-freezing properties of hydrogels.

Methods	Hydrogels Network	Antifreezing Point/°C		References
		Before Enhancement	After Enhancement	
Introducing soluble ions in solvents	Integrating KCl, NaCl or CaCl_2_ into hydrogels	−48.4	−17.3	[24]
	Integrating LiCl into polyacrylamide-cellulose nanofibrils (CNF) double-network hydrogels	−80	−20	[25]
	Integrating NH_4_Cl into Ca-alginate/polyacrylamide hydrogels with betaine and proline	−40	N/A	[26]
	Integrating LiCl and KCl into carrageenan (CG)/polyacrylamide based double-network hydrogels	−40	N/A	[27]
	Integrating CaCl_2_ into polyacrylamide-alginate double-network hydrogels	−57	−3	[17]
	Integrating KCl into zwitterionic poly(ionic liquid) by copolymerization of 1-vinyl-3-(carboxymethyl)-imidazole and acrylamide (AAM)	−20	−3	[28]
	Integrating LiCl into poly(N-hydroxyethyl acrylamide-co-utanediol) (P(HEA-co-BD)) double-network hydrogels	−85.6	−17.1	[29]
	Integrating Fe_2_(SO_4_)_3_ into chitosan-poly(acrylic acid-co-acrylamide) (CS/P(AA-co-AAM)) double-network hydrogels	−20	0	[30]
Introducing organic solvent into hydrogels	Ca-alginate/polyacrylamide organohydrogels in glycerol, glycol, sorbitol or mixtures	−70	N/A	[31]
	Polymer networks with reversible crosslinked photoresponsive Ru-thioether coordination bonds in glycerol/water (H_2_O) binary solvent	−20	0	[19]
	Incorporating conductive poly(3,4-ethylenedioxythiophene)/polystryrene sulfonate (PEDOT/PSS) polymers into poly(vinyl alcohol) (PVA) networks in ethylene glycol (EG)/H_2_O binary solvent	−55	−10	[14]
	Crosslinking polyacrylic acid (PAA), PVA and borax in EG/H_2_O binary solvent	−90	−5	[32]
	Hydrogen-bonded supramolecular organohydrogels by gelation of PVA in glycerol/H_2_O binary solvent	−78.5	−50	[18]
	Polymerization with sodium methacrylate (MAANa) and [2-(methacryloyloxy)ethyl] trimethyl ammonium chloride in glycerol/H_2_O binary solvent	−20	−7.7	[33]
	Integrating HEA monomers in glycerol/H_2_O binary solvent	−30	−4	[34]
	Integrating tannic acid-carbon nanotubes (TA-CNT) into PVA matrix in glycerol/H_2_O binary solvent	−30	N/A	[35]
	Montmorillonite (MMT)/PVA organohydrogels in dimethyl sulfoxide (DSMO)/H_2_O binary solvent	−50	N/A	[36]
	Integrating high concentration LiTFSI into chitosan hydrogels in DMSO/H_2_O binary solvent	−20	N/A	[37]
	Organohydrogels by sol-gel transition of PVA and CNF in DMSO/H_2_O binary solvent	−70	−20	[38]
	Poly(vinyl alcohol), phenylboronic acid-grafted alginate and polyacrylamide organohydrogels in reduced graphene oxide (rGO)-contained EG/H_2_O binary solvent	−40	N/A	[39]
Introducing soluble ions in solvents and organic solvent into hydrogels	Integrating ionic compounds (ZnCl_2_/CaCl_2_) and glycerol into cellulose hydrogel networks	−70	−60	[40]
	Integrating LiCl into PVA networks in glycerol/H_2_O binary solvent	−60	N/A	[41]
	Integrating nanolignin and AlCl_3_ into PVA matrix in EG/H_2_O binary solvent	−62.6	−16	[13]
	Integrating ZnSO_4_ into PVA/polyacrylamide (PAM) double-network hydrogels in EG/H_2_O binary solvent	−50	N/A	[42]
	Integrating LiClO_4_ into hydroxypropyl cellulose (HPC)/PVA double networks in glycerol/H_2_O binary solvent	−40	N/A	[43]
Introducing ionic liquids as solvents to form ionogels	Ionogels by photoinitiated polymerization of 2,2,2-trifluoroethyl acrylate (TFEA) and AAM in hydrophobic 1-ethyl-3-methylimidazolium bis(trifluoromethanesulfonyl)imide ([EMIM][TFSI]	−30	N/A	[44]
	Click-ionogels with poly(1-butyl-3-vinyl imidazolium fluoborate) (PIL-BF_4_) and benzene tetracarboxylic acid (BTCA) ionic crosslinked network and thiol-ene clicknetwork	−75	0	[45]
	Ionogels by hydrogen bonding between poly (ethyl acrylate) based elastomer and 1-ethyl-3-methylimidazolium bis(trifluoromethylsulfonyl)imide ([C_2_mim][NTf_2_]) based ionic liquids	−70	N/A	[21]
	Ionogels by locking the ionic liquid of 1-ethyl-3-methylimidazolium dicyanamide ([EMIm][DCA]) into charged poly(2-acrylamido-2-methyl-1-propanesulfonic acid) (PAMPS)-based double networks	−70	N/A	[46]
	Free radical polymerization based on precursors of N,N′-methylenebisacrylamide (MBAA), N,N-dimethylacrylamide (DMAA) and 2,2-diethoxyacetophenone (DEAP) in 1-ethyl-3-methylimidazolium tetrafluoroborate (EMIMBF_4_) ionic liquid	−40	N/A	[20]
Modifying polymer network	EG based waterborne anionic polyurethane acrylates (waPUA)/PAM based double crosslinked hydrogels	−20	N/A	[22]
	Free-radical polymerization of an alkalified PAA hydrogel equipped with copious carboxyl groups on the polymer chain in KOH solution	−25	N/A	[23]
	Introducing antifreeze protein of AFPS into the chemical crosslinking network copolymerized with AAM and 2-acrylamide-2-methylpropanesulfonic acid (AMPS)	−10	−10.5	[47]
	Supramolecular DMAA and 2-(N-ethylperfluorooctane sulfonamido) ethyl acrylate (FOSA) copolymer hydrogels with water confined by hy-drophobic FOSA nanodomain crosslinks	−68	N/A	[48]
	Supramolecular hydrogels formed from copolymers of 2-hydroxyethyl acrylate (HEA) and 2-(N-ethylperfluorooctane sulfonamido) ethyl methacrylate (FOSM)	−145	0	[49]

**Table 2:** Typical polymers used in thermoresponsive hydrogels.

Type	Polymers	Abbreviation	Critical Solution Temperature/°C	References
LCST	Poly(ethylene glycol)	PEG	106–115	[79]
	Poly(propylene glycol)	PPG	10–40	[80]
	Poly(vinyl alcohol)	PVA	125	[81]
	Poly(N-isopropylacrylamide)	PNIPAM	32	[82,83]
	Poly(N,N′-diethylacrylamide)	PDEAM	25	[84]
	Poly(N-ethylmethacrylamide)	PNEMAM	58	[84]
	Poly(N-vinylisobutyramide)	PNVIBAM	39	[85]
	Poly(methyl vinyl ether)	PMVE	34	[86]
	Poly(2-ethoxyethyl vinyl ether)	PEOVE	20	[87]
	Poly(N-vinyl caprolactam)	PNVCa	30–50	[88,89]
	Poly(ethylene oxide)-poly(propyleneoxide)-poly(ethylene oxide)	PEO-PPO-PEO	30	[90]
UCST	Poly (N-acryloylglycinamide)	PNAGA	18	[91]
	Poly(3-imidazolyl-2-hydroxypropyl methacrylate)	PiGMA	37	[92]
	Poly(acrylamide-co-acrylonitrile)	P(AAM-co-AN)	32	[93]
	Poly(allylamine)-co-poly(allylurea)	PAU	65	[94]
	Polysulfobetaine	PBET	40	[95]
	Poly(sulfobetaine-co-sulfabetaine)	P(SB-co-ZB)	30–45	[96]

## Data Availability

Not applicable.
